# Effect of archwire plane and archwire size on anterior teeth movement in sliding mechanics in customized labial orthodontics: a 3D finite element study

**DOI:** 10.1186/s12903-022-02066-9

**Published:** 2022-02-10

**Authors:** Jianhua Wu, Xiaoting Wang, Yiyang Jiang, Zichen Wu, Qun Shen, Yucheng Chen, Qianjiao Meng, Niansong Ye

**Affiliations:** 1 Department of Orthodontics, Hefei Stomatological Hospital, Annhui, China; 2 Department of Orthodontics, Shanghai Xuhui District Dental Center, Shanghai, China; 3 Wake Forest University, Winston-Salem, North Carolina USA; 4 College of Stomatology, Annhui Medical University, Annhui, China; 5 Private Practice, Shanghai Huaguang Dental Clinic, 6C, No.201, Lane 3215, Hongmei Road, Shanghai, China

**Keywords:** Customized labial orthodontics, Archwire plane, Archwire size, Sliding mechanics, Finite element analysis

## Abstract

**Background:**

The aim of this study was to evaluate anterior teeth movement with different archwire planes and archwire sizes during space closure with and without miniscrew in sliding mechanics.

**Methods:**

A 3D finite element method was applied to simulate anterior teeth retraction with and without miniscrew and power arm. Initial displacements and pressure stresses of periodontal tissue in anterior teeth were calculated after the teeth were applied with retraction forces with different archwire planes and archwire sizes.

**Results:**

High archwire plane showed better torque control of anterior teeth in both sliding mechanics. With intramaxillary retraction, anterior teeth showed lingual tipping and extrusion movement, whereas larger-size archwires did not reduce it. In miniscrew sliding mechanics, anterior teeth showed labial tipping and intrusion movement. Compared with intramaxillary retraction, the retraction force produced less pressure stress on periodontal tissue in miniscrew sliding mechanics with long power arm.

**Conclusions:**

Higher archwire plane is conducive to anterior teeth torque control. In order to achieve the bodily movement of the anterior teeth during space closure, it is more important to choose the appropriate method (miniscrew sliding mechanics with long power arm), instead of increasing the size of the archwire.

**Supplementary Information:**

The online version contains supplementary material available at 10.1186/s12903-022-02066-9.

## Background

With the developments of three-dimensional imaging and manufacturing technology, the customized orthodontic appliances improves treatment outcomes effectively and achieve the digital design accurately [1, 2]. The customized bracket system would allow orthodontists to provide high-quality treatment in less time, with fewer appointments and reduced chair time [3, 4].

Different from the traditional straight wire system that uses the center of the clinical crown (FA point) to position the bracket, the customized appliances position the brackets based on the archwire plane. First, the digital setup model was designed in the customized appliances design software system according to the orthodontist’s prescription. Second, an arch form plane is set which interacts the buccal surface of each tooth, and the customized straight wire is then designed on this plane. Third, the customized brackets and tubes are positioned on the buccal surface of the teeth along the archwire. Finally, indirect-bonding transfer jigs are designed based on the position of the brackets [5]. Therefore, the position of the different archwire planes determines the brackets position. In the digital setup process, there are some regulations that should be followed to facilitate the clinical treatment. For example, in the design of a deep bite case, technicians will often design the anterior archwire plane closer to the incisal margin, in order to get more anterior teeth intrusion during the alignment and leveling phase. For patients with an open bite, the archwire plane is often designed closer to the gingiva in order to get more anterior teeth extrusion during the alignment and leveling phase. In addition, for adolescents, the clinical crown height is usually insufficient so that the brackets in anterior segment are often placed closer to incisal margin in order prevent any influence on gingival health. The archwire plane is not consistent with the traditional plane made up of FA points in various conditions. Meanwhile, diverse archwire planes will lead to different effects on anterior teeth movement under orthodontic force [6].

In premolar extraction treatment, common options for space closure used by orthodontist are traditional sliding mechanics. In the severe protrusion, the miniscrews is commonly used to obtain maximum anterior segment retraction [7, 8]. And as the change of miniscrew position, the different biomechanics can be produced. When the low-positioned miniscrew is combined with the short power arm, the resulting force line is lower than center of resistance (CR) of anterior segment, resulting in the lingual tipping of the anterior teeth. When the high-positioned miniscrew is combined with long power arm, the force line passes through the CR, which can achieve the bodily movement of anterior teeth [9]. The traditional sliding mechanics without miniscrew extraction are typically used in the case of moderate anchorage [10], which are usually generated by coil spring or elastic chain from power arm of anterior segment to the hook of second molar. However, as the force line below the CR of both anterior and posterior segment, the vertical bowing effect (torque loss and extrusion of the anterior) is occurred [11]. In space closure, most studies suggested that in order to control the torque of the anterior teeth, a larger size archwire is preferred [12, 13]. Previous study also stated that miniscrew with a long power arm would facilitate bodily movement of anterior teeth [14, 15]. However, the impact of the archwire plane and archwire sizes on anterior teeth movement during space closure in sliding mechanics has not been clarified.

Finite element analysis (FEA), an effective mathematical method, is widely used to quantify and visualize orthodontic teeth movement and stress of periodontal tissue in three-dimensional situation [16, 17]. In the present study, 3D finite element models were established to compare the effect of the archwire plane and archwire size on anterior teeth movement during space closure in two different sliding mechanics.

## Methods

**Fig. 1:**
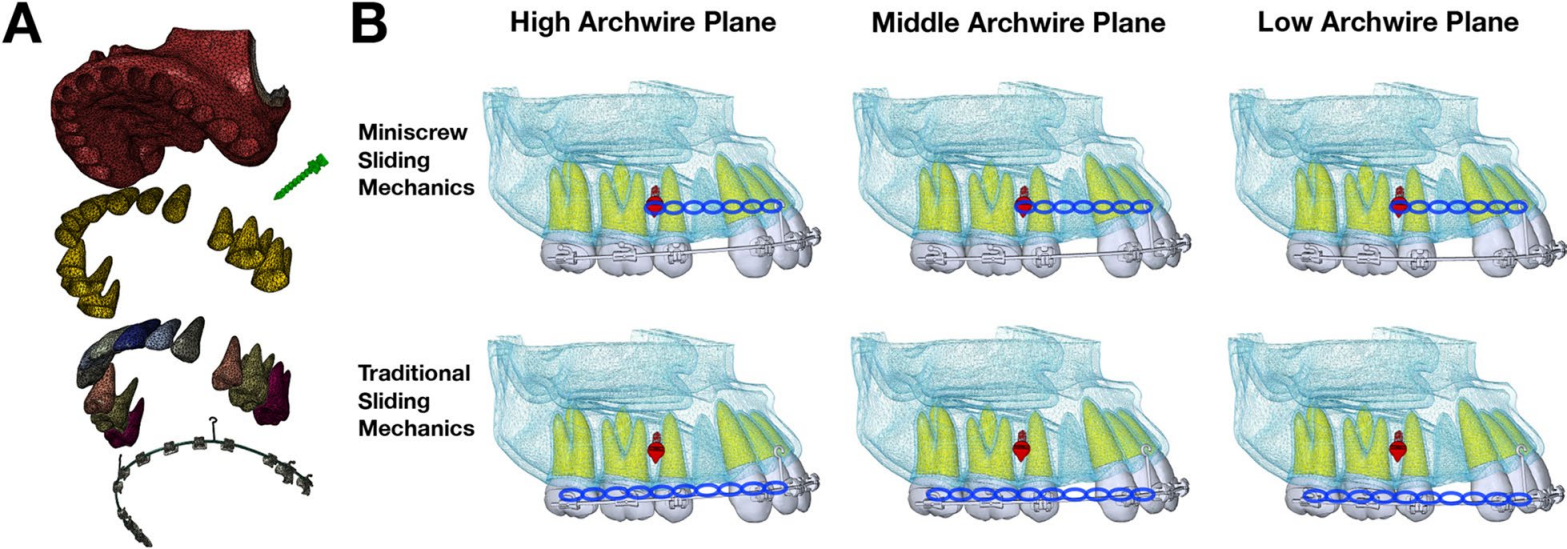
**A** 3D finite element model, including alveolar bone, miniscrew, PDL, dentition, bracket and archwire. **B** Miniscrew sliding mechanics and traditional sliding mechanics with low, middle and high archwire plane

Cone-beam computed tomography (CBCT) images of the maxillary bone and teeth, taken with a CBCT scanner (KaVo Dental, Biberach, Germany), were saved as DICOM data and exported. Alveolar bone and upper teeth models were 3D reconstructed in the Mimics 10.01 software (Materialize Software, Leuven, Belgium). 0.2 mm thickness periodontal ligament (PDL) model was raised from the roots. And all elements including teeth, PDL, alveolar bone, brackets, archwires, power arms and miniscrews were assembled and converted into Abaqus software (version 6.14, Dassault System, France) (Fig. 1A). The elements were constructed as 2nd order Tetra. The materials properties were defined as teeth (Young's modulus, 20Gpa; Poisson ratio, 0.3), PDL (Young's modulus, 0.068 Gpa; Poisson ratio, 0.49), alveolar bone (Cortical bone, Young's modulus, 13.4Gpa; Poisson ratio, 0.38. Cancellous bone, Young's modulus, 7.8Gpa; Poisson ratio, 0.38), bracket (Young's modulus, 214Gpa; Poisson ratio, 0.3), archwire (Young's modulus, 200Gpa; Poisson ratio, 0.3) and power arms (Young's modulus, 200Gpa; Poisson ratio, 0.3). And the miniscrews were constructed with the following parameters (Ormco VectorTAS, 8 mm; Young's modulus, 103Gpa; Poisson ratio, 0.33). The above material properties used according to previous studies [18, 19]. The interfaces between alveolar bone and PDL, PDL and tooth, tooth and bracket, archwire and power arm were bonded relationship. The surface contact was applied to define the relationship between archwire and brackets, assuming a friction coefficient of 0.2 according to previous studies [14, 20].

In this study, a total of 18 finite element models were established based on 3 different archwire planes (low, middle and high), 3 archewire sizes (stainless steel: 0.018 × 0.025-inch, 0.019 × 0.025-inch and 0.021 × 0.025-inch), and 2 kinds of sliding mechanics (with and without miniscrews). The 0.018 × 0.025-inch model with miniscrews was comprised of a total of 337,229 elements (624,179 nodes) and one without miniscrews was comprised of 334,597 elements (619,015 nodes). The 0.019 × 0.025-inch and 0.021 × 0.025-inch models had similar number. Archwire planes were divided into three groups: low archwire plane (LAP), middle archwire plane (MAP) and high archwire plane (HAP). MAP was defined as a connection of FA points of anterior teeth and posterior teeth, which was also the position of the bracket. The difference between LAP, HAP from MAP was found in anterior teeth. In LAP, the position of bracket was in the midpoint between incisal margin and FA points of anterior teeth. While in HAP, the location of bracket is in the midpoint between gingiva margin and FA points of anterior teeth (Fig. 1B).

The power arms were located bilaterally at the midpoint between the lateral incisors and the canines, and the miniscrews were placed bilaterally between the second premolars and the first molars. The vertical distance between the center of the miniscrew and the archwire plane was 8 mm. The length of power arm in the middle archwire plane was defined to be 8 mm. In order to unify the direction of the retraction force parallel to the occlusion plane in miniscrew sliding mechanics, the lengths of the power arm in high or low archwire plane were adjusted to be consistent with the upper end of the power arm in the middle archwire plane. In the model with miniscrews, the retraction force (1.5 N) was applied from the hook of power arm to the miniscrews on each side. In model without miniscrew, the retraction force was applied from the connection point of power arm to the buccal tube of second molar on each side, which was also known as traditional sliding mechanics (Fig. 1B). During the retraction, each tooth acted separately. The upper and back parts of the maxilla were set as the boundary region to limit elements movements in all directions. The customized bracket slot was set to be 0.022 × 0.028-inch.

A coordinate system with X, Y, and Z axis was applied on each tooth, the X axis represented the mesio-distal direction (− mesial, + distal), the Y axis represented the vertical direction (− occlusal, + apical), and the Z axis represented the bucco-lingual direction (− lingual, + buccal). The initial displacement of the reference nodes of the anterior teeth and the pressure stresses produced onto the teeth, PDL and alveolar bone were measured by using the visualization results of the 3D initial teeth movements. And the displacements and pressure stresses were compared among 3 different archwire planes, 3 archewire sizes, and 2 kinds of sliding mechanics to facilitate realization of controllable teeth movement during space closure in customized orthodontics.

## Results

**Fig. 2:**
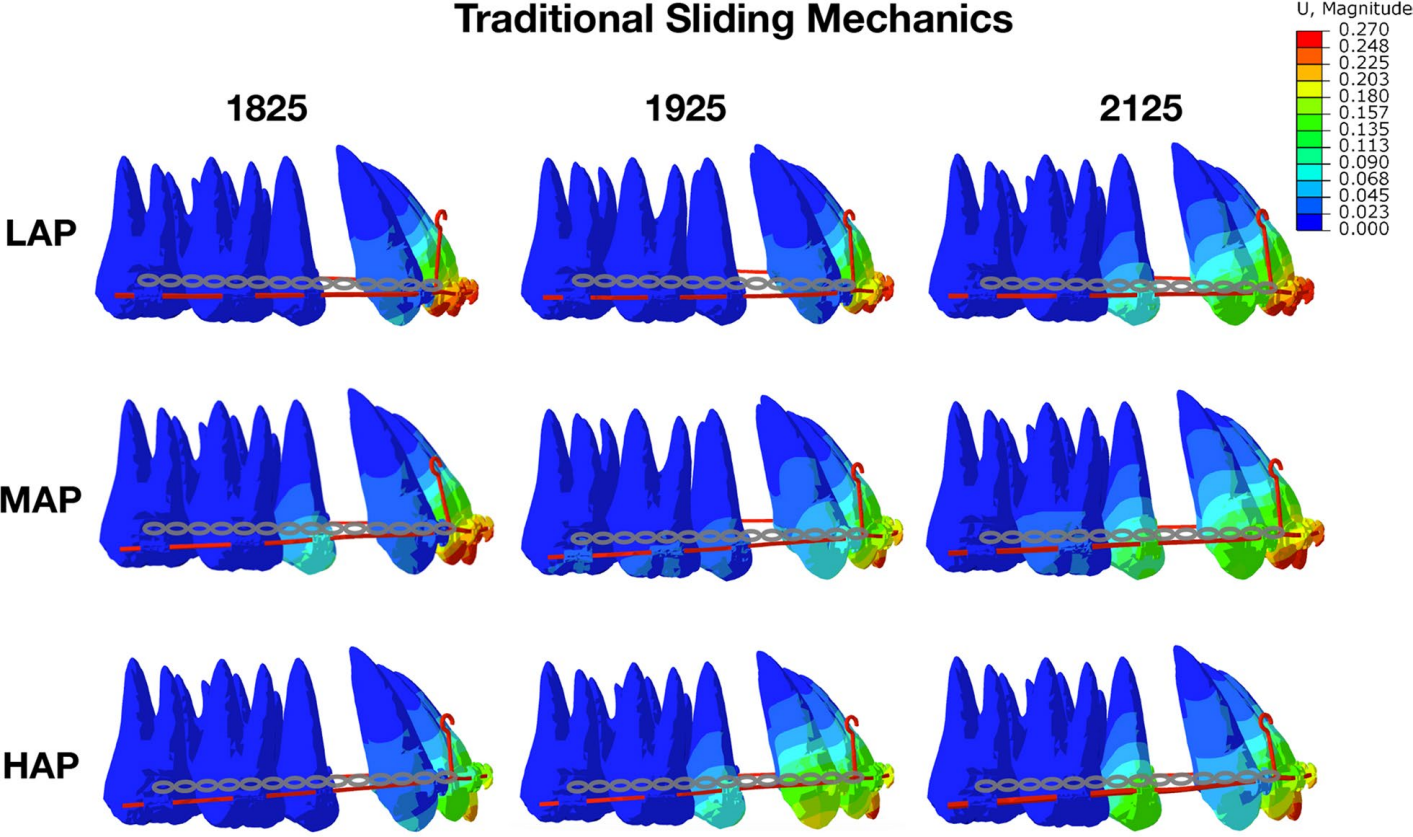
Initial movement of maxillary anterior teeth and archwire deformation in traditional sliding mechanics. The lingual tipping was observed in all archwire sizes, but fewer in high archwire plane than low archwire plane. Unit: mm

**Fig. 3:**
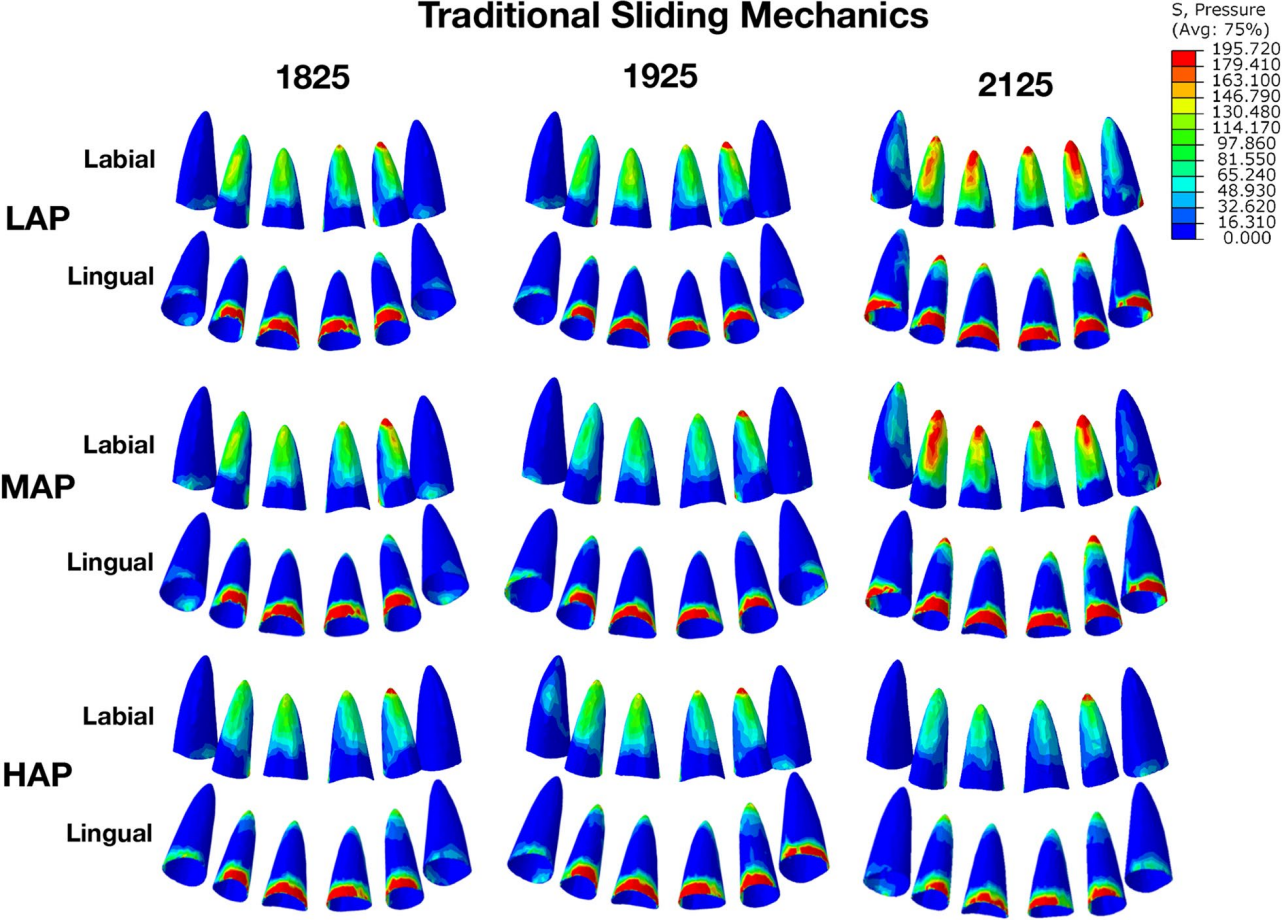
The pressure stresses on PDL in traditional sliding mechanics. The pressure stress focused on the labial apical area and the lingual neck area, indicating the lingual tipping movement of incisors. Unit: KPa

In traditional sliding models, the lingual tipping (incision-apex) was observed in all archwire sizes (Fig. 2), and no differences were observed between small and large-size archwires. However, with high archwire plane, extrusion and lingual tipping of the central and lateral incisors were lower than those with low and middle archwire planes (Table 1). The pressure stresses were observed on the root surfaces, PDL and alveolar bone in Table 2. For PDL in incisors, the pressure stress focused on the labial apical area and the lingual neck area (Fig. 3). As the archwire size increased, the pressure stresses of PDL and alveolar bone were also increasing. In contrast, as the increasing of archwire plane (from LAP to HAP), the pressure stresses were decreased (Table 2). In addition, after applying retraction force, the form of archwire changed at the connection point of power arm according to the video of 3D initial teeth movements. The effect of retraction force mainly manifested as the lingual tipping and extrusion of the central and lateral incisors (see Additional file 1), and it is consistent with the data presented in the Table 2.

**Table 1:** Initial displacement of reference nodes in traditional sliding mechanics (mm)

			X			Y			Z		
			Low	Middle	High	Low	Middle	High	Low	Middle	High
1825	Central incisor	Incision	0.0198	0.0294	0.0182	− 0.0441	− 0.0487	− 0.0418	− 0.2488	− 0.2360	− 0.1987
		Apex	0.0044	− 0.0009	0.0041	− 0.0390	− 0.0021	− 0.0092	− 0.0105	− 0.0112	− 0.0137
	Lateral incisor	Incision	0.0136	0.0437	0.0250	− 0.0227	− 0.0151	− 0.0128	− 0.2481	− 0.2490	− 0.1461
		Apex	0.0081	0.0007	0.0075	− 0.0159	− 0.0072	− 0.0041	− 0.0029	− 0.0035	− 0.0087
	Canine	Incision	0.0146	0.0348	0.0318	− 0.0267	− 0.0107	− 0.0251	− 0.0151	− 0.0202	− 0.0039
		Apex	0.0053	− 0.0004	0.0020	− 0.0043	− 0.0033	− 0.0041	− 0.0032	− 0.0030	− 0.0039
1925	Central incisor	Incision	0.0540	0.0195	0.0137	− 0.1226	− 0.0496	− 0.0387	− 0.2552	− 0.2310	− 0.2000
		Apex	− 0.0006	0.0019	0.0027	− 0.0104	− 0.0038	− 0.0024	− 0.0100	− 0.0128	− 0.0149
	Lateral incisor	Incision	0.0278	0.0539	0.0842	− 0.0309	− 0.0067	− 0.0103	− 0.2128	− 0.2216	− 0.1660
		Apex	0.0043	0.0057	0.0055	− 0.0126	− 0.0065	− 0.0079	− 0.0047	− 0.0054	− 0.0080
	Canine	Incision	0.0205	0.0859	0.0271	− 0.0338	− 0.0107	− 0.0139	− 0.0118	− 0.0185	− 0.0540
		Apex	− 0.0023	0.0026	0.0025	− 0.0057	− 0.0035	− 0.0027	− 0.0032	− 0.0047	− 0.0030
2125	Central incisor	Incision	0.0458	0.0163	0.0146	− 0.1016	− 0.1005	− 0.0381	− 0.2560	− 0.2331	− 0.2245
		Apex	− 0.0033	0.0063	0.0030	− 0.0037	− 0.0111	− 0.0024	− 0.0161	− 0.0191	− 0.0169
	Lateral incisor	Incision	0.0354	0.0452	0.0386	− 0.0378	− 0.0551	− 0.0106	− 0.2313	− 0.1943	− 0.1764
		Apex	0.0094	0.0093	0.0063	− 0.0228	− 0.0205	− 0.0118	− 0.0079	− 0.0125	− 0.0099
	Canine	Incision	0.0409	0.0356	0.0544	− 0.0338	− 0.0345	− 0.0089	− 0.0050	− 0.0196	− 0.0215
		Apex	0.0016	0.0053	0.0068	− 0.0027	− 0.0145	− 0.0028	− 0.0065	− 0.0061	− 0.0046

X: − mesial, + distal; Y: − occlusal, + apical; Z: − lingual, + buccal

**Table 2:** The pressure stress of reference nodes in traditional sliding mechanics (KPa)

		1825			1925			2125		
		Low	Middle	High	Low	Middle	High	Low	Middle	High
Tooth	Central incisor	88.96	39.71	65.03	76.37	41.29	32.01	89.86	66.53	35.09
	Lateral incisor	75.06	47.66	88.07	112.52	46.96	36.74	106.11	144.50	45.67
	Canine	28.25	26.22	91.99	52.18	28.92	28.08	103.13	86.48	15.85
Alveolar bone	Central incisor	94.92	47.85	91.48	181.88	73.14	53.05	106.04	98.02	90.19
	Lateral incisor	80.50	119.16	121.51	121.77	61.47	64.31	133.83	146.69	59.89
	Canine	46.99	20.83	107.81	69.52	42.18	20.47	119.10	127.55	27.45
PDL	Central incisor	255.52	123.47	190.33	219.97	103.00	88.30	236.76	215.82	102.20
	Lateral incisor	188.08	104.35	236.47	341.14	140.49	134.17	333.84	396.91	139.85
	Canine	24.00	62.23	112.76	68.88	31.11	30.43	138.40	113.17	18.66

**Fig. 4:**
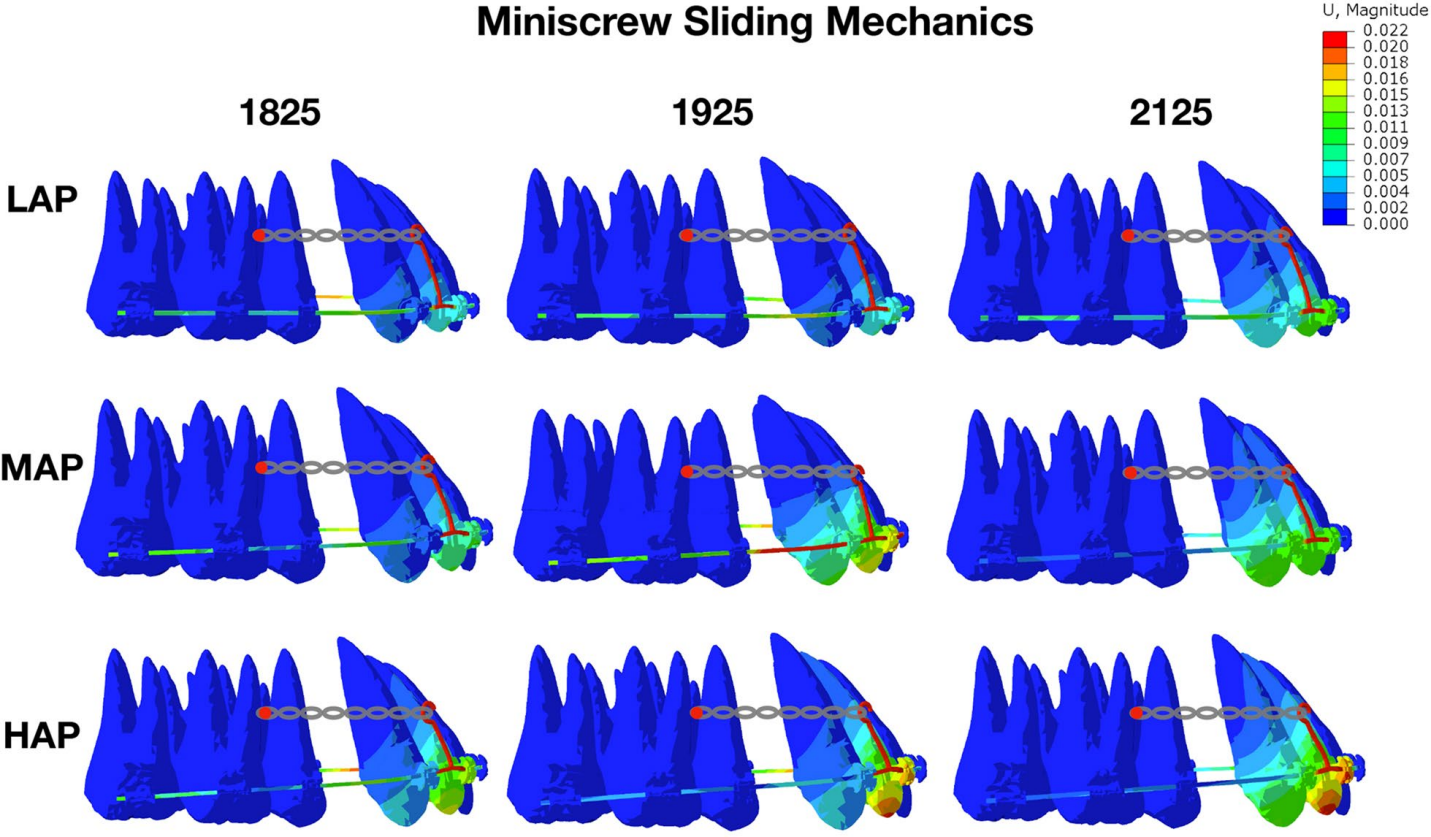
Initial movement of maxillary anterior teeth and archwire deformation in miniscrew sliding mechanics. The labial tipping was observed in all archwire sizes and archwire planes. Unit: mm

**Fig. 5:**
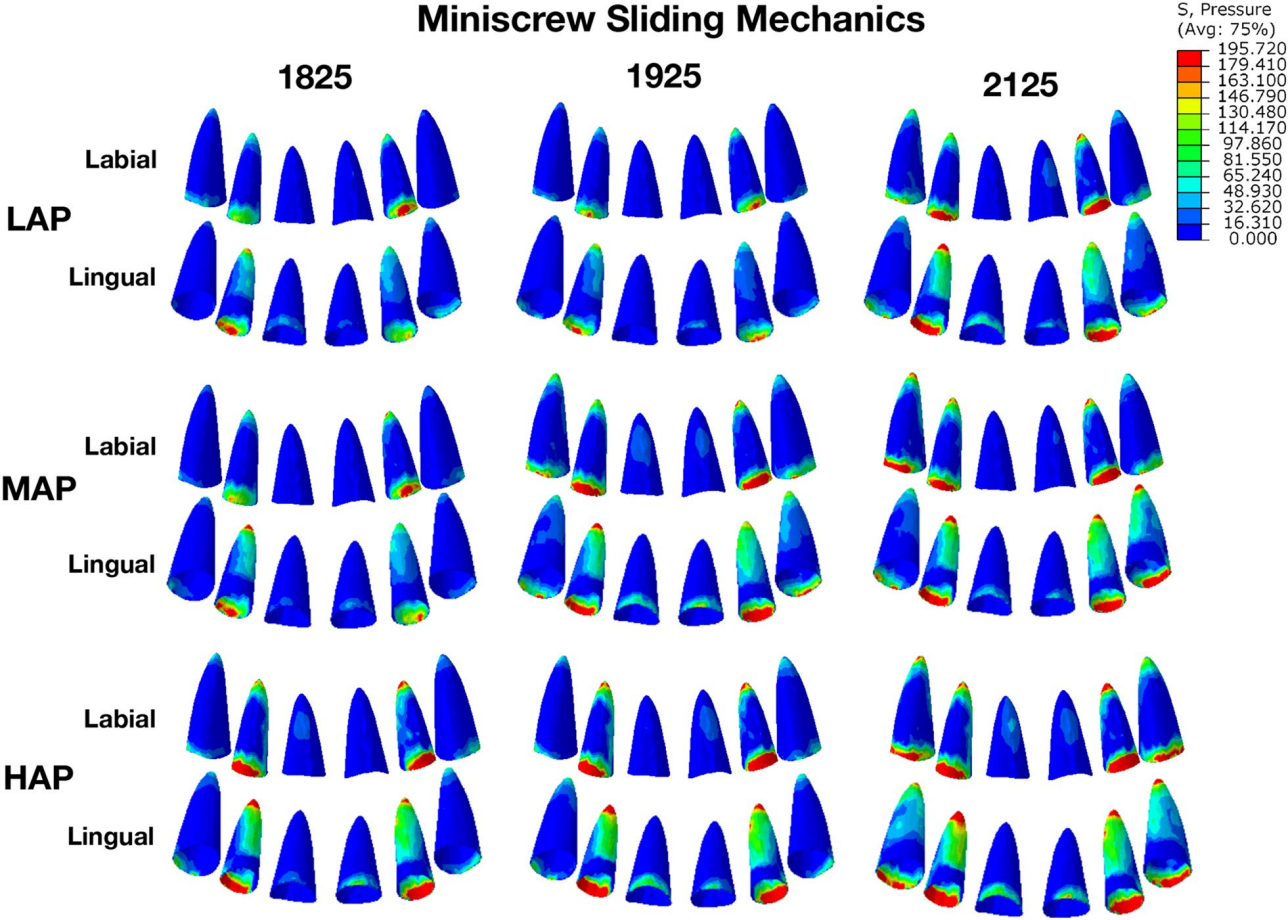
The pressure stresses on PDL in miniscrew sliding mechanics. The pressure stress focused on the lingual apical area and the labial neck area, indicating the labial tipping movement of incisors. Unit: KPa

In miniscrew sliding models, opposite to traditional sliding models, the labial tipping was observed in all conditions (Fig. 4). As for lateral incisor, the amount of labial tipping increased with the increase of archwire sizes and archwire planes. However, the tendency of labial tipping in central incisor and canine was not obvious as lateral incisor. As the archwire sizes and archwire planes increased, the intrusion amount of all anterior teeth also increased (Table 3). Consistent with the data in Table 4, the pressure stresses on PDL in Fig. 5 focused on the labial neck area and the lingual apical area, which indicated the labial tipping. The pressure stresses on PDL and alveolar bone increased with the increasing of archwire sizes and archwire planes (Table 4). Besides, in miniscrew sliding models, the deformation of archwire form started from the hook of power arm, leading to a counterclockwise twist of archwire (see Additional file 2), which might result in labial tipping. Since the twisted archwire located between the lateral incisors and canines, the tipping amount of central incisors was relatively low.

**Table 3:** Initial displacement of reference nodes in miniscrew sliding mechanics (mm)

			X			Y			Z		
			Low	Middle	High	Low	Middle	High	Low	Middle	High
1825	Central incisor	Incision	0.0003	0.0004	0.0000	0.0013	0.0021	0.0042	0.0028	0.0018	0.0014
		Apex	0.0001	0.0001	0.0001	0.0020	0.0023	0.0037	− 0.0006	− 0.0007	− 0.0010
	Lateral incisor	Incision	0.0067	0.0046	0.0064	0.0054	0.0064	0.0105	0.0341	0.0393	0.0680
		Apex	0.0001	0.0002	0.0005	0.0031	0.0039	0.0064	− 0.0004	0.0006	0.0012
	Canine	Incision	0.0062	0.0061	0.0092	0.0036	0.0044	0.0068	0.0103	0.0050	0.0016
		Apex	− 0.0002	− 0.0002	− 0.0002	0.0015	0.0018	0.0028	0.0005	− 0.0005	0.0008
1925	Central incisor	Incision	0.0001	0.0011	0.0014	0.0020	0.0002	0.0027	0.0013	0.0018	0.0046
		Apex	0.0001	0.0001	0.0003	0.0017	0.0008	0.0038	− 0.0006	− 0.0003	0.0011
	Lateral incisor	Incision	0.0027	0.0041	0.0097	0.0042	0.0024	0.0126	0.0280	0.0472	0.0716
		Apex	0.0002	0.0000	0.0009	0.0027	0.0013	0.0071	− 0.0003	0.0003	0.0013
	Canine	Incision	0.0055	0.0026	0.0100	0.0036	0.0016	0.0077	0.0040	0.0050	0.0017
		Apex	0.0002	− 0.0001	0.0002	0.0014	0.0008	0.0032	− 0.0004	0.0002	0.0009
2125	Central incisor	Incision	0.0010	0.0011	0.0005	0.0031	0.0036	0.0046	0.0015	0.0006	0.0031
		Apex	0.0002	0.0002	0.0004	0.0030	0.0039	0.0052	− 0.0010	0.0013	0.0016
	Lateral incisor	Incision	0.0076	0.0023	0.0048	0.0073	0.0104	0.0151	0.0465	0.0747	0.0812
		Apex	0.0003	0.0007	0.0008	0.0049	0.0066	0.0091	− 0.0005	0.0006	0.0011
	Canine	Incision	0.0115	0.0211	0.0278	0.0067	0.0100	0.0141	0.0019	0.0034	0.0035
		Apex	− 0.0001	0.0001	− 0.0001	0.0027	0.0043	0.0051	0.0009	0.0016	0.0018

X: − mesial, + distal; Y: − occlusal, + apical; Z: − lingual, + buccal

**Table 4:** The pressure stress of reference nodes in miniscrew sliding mechanics (KPa)

		1825			1925			2125		
		Low	Middle	High	Low	Middle	High	Low	Middle	High
Tooth	Central incisor	1.79	1.75	2.08	1.29	0.90	2.66	2.04	2.42	3.01
	Lateral incisor	8.57	11.12	21.17	7.67	4.61	27.08	13.16	15.60	40.14
	Canine	2.87	3.19	3.91	2.72	1.92	4.52	5.37	9.82	10.61
Alveolar bone	Central incisor	2.37	1.99	2.99	1.26	1.68	4.15	2.41	3.38	4.58
	Lateral incisor	5.20	7.71	15.41	5.11	2.92	16.91	9.94	11.19	18.23
	Canine	2.91	3.51	5.57	2.87	1.65	5.96	5.98	10.24	11.09
PDL	Central incisor	1.70	1.81	4.66	2.23	2.06	6.46	3.51	4.53	7.04
	Lateral incisor	15.35	15.47	40.56	17.01	7.62	45.10	20.90	31.15	46.87
	Canine	3.10	2.62	7.20	5.29	2.50	8.75	10.01	18.30	21.31

Compared between two sliding mechanics, the pressure stresses of PDL and alveolar bone in traditional sliding mechanics were greater than those in miniscrew sliding mechanics. The retraction effect of traditional sliding mechanics was stronger, but the movement of anterior teeth manifested as lingual tipping and extrusion. On the contrary, in miniscrew sliding mechanics, all the anterior teeth displayed labial tipping and intrusion movement.

## Discussion

In customized orthodontic treatment, the customized bracket is positioned according to the virtual archwire plane. The setup of archwire plane is partly based on the height of the clinical crown, occlusion conditions and aimed position, and etc. For example, short clinical crown and over bite may lead to a low archwire plane. On the contrary, open bite will lead to a high archwire plane. In premolar extraction treatment, since different archwire planes have different distances to CR of the anterior teeth, the archwire plane will produce dissimilar effects on anterior teeth. Based on the results of our retrieval for previous relevant studies, it is currently the first study on the original concept. According to this study, the archwire plane did affect the anterior teeth movement, especially in intramaxillary retraction. With the decrease of archwire plane, the anterior teeth showed greater lingual tipping and extrusion during retraction, since the moment arm (vertical distance from CR to line of retraction force) of high archwire plane was shorter than that of low archwire plane. These findings suggested that higher archwire plane could reduce lingual tipping and extrusion of anterior teeth rather than avoid it in intramaxillary retraction.

The research from Kojima et al. stated that body movement of anterior teeth could be achieved with 8 mm power arm and high-position miniscrew [13]. Similar results were found in our study: in miniscrew sliding mechanics with long power arm, the anterior teeth moved almost bodily. And the archwire planes had limited effect on the tipping of the anterior teeth since the direction of the retraction force were the same among different archwire planes. Besides, in miniscrew sliding mechanics the intrusion of anterior teeth was found in all archwire planes, and higher archwire planes showed greater effect on the intrusion of anterior teeth. Since the placement of the miniscrew was apical to the CR [21], the anterior dentition was exposed to vertical component of retraction force [22]. We found that in the in miniscrew sliding mechanics, the effect of retraction was manifested as the deformation of the hook and the uplift of archwire. And the uplift of the archwire, acted as a cantilever effect, would produce an intrusion force on the anterior teeth [14, 20]. We speculated that due to the relatively longer length of the hook in LAP, part of the force was consumed by the deformation of the hook. As a result, the intrusion effect of the archwire on the anterior teeth might have been weakened in LAP.

Previous FEA studies mostly focused on tooth movement, and our research innovatively compared the pressure stresses between different archwire planes in both sliding models, and found the exact opposite pattern. In miniscrew sliding model, the pressure stresses increased with the increasing of archwire plane. This may be due to the greater cantilever effect in higher archwire plane. The pressure stresses decreased with the increasing of archwire plane in traditional sliding model. The reason might be that in intramaxillary retraction, the retraction force acted directly on the archwire. Also, the higher plane reduced the tipping movement of anterior teeth due to shorter moment arm, which led to a more uniform force on the teeth and periodontal tissue. Compared between the two different sliding mechanics, we could clearly find that the pressure stresses on teeth and periodontal tissue in miniscrew sliding mechanics was lower than in intramaxillary retraction. The research from Zhong et al. stated heavy pressure in PDL generated by orthodontic forces lead to larger root resorption volumes than light pressure [23]. These results indicated that a long power arm combined with miniscrews could produce even pressure on PDL during retraction, which were expected in clinical treatment.

Torque loss of the anterior teeth caused by the retraction force was reflected as vertical bowing effects. Previous studies showed that increased archwire size would tipping in miniscrew sliding mechanics [12, 13]. However, we have not retrieved references on the influence of archwire size on the torque of the anterior teeth during intramaxillary retraction. In our research, uncontrolled lingual crown tipping of the anterior teeth was observed in intramaxillary retraction as pervious study [14]. The increasing archwire sizes didn’t rescue this vertical bowing effects, but produce higher the pressure stresses. These findings indicated that in intramaxillary retraction, it was ineffective to reduce vertical bowing effects in anterior segment by increasing size of the archwire.

The above FEA results provided some keynotes to clinical practice. First, archwire plane did affect the movement and pressure stresses of anterior teeth. Second, lower archwire plane could increase lingual tipping and extrusion movement, and increase the pressure stresses of periodontal tissue of anterior teeth, which might add the risk of root resorption during intramaxillary retraction. Finally, high-positioned miniscrew with long power arm could achieve bodily retraction of anterior teeth. It should be noted that the initial displacement in the digital model may not accurately reproduce clinical orthodontic movements. In the future, a clinical trial will be recommended to verify these FEA conclusions.

## Conclusions

Sliding mechanics with and without miniscrew provoke different anterior teeth movements and stress patterns during space closure. Higher archwire plane is conducive to anterior teeth torque control. In order to achieve the bodily movement of the anterior teeth during space closure, it is more suggested to choose the appropriate method (miniscrew sliding mechanics with long power arm), rather than by simply increasing the size of the archwire.

## Supplementary Information

**Additional file 1**: Video for virtual teeth movement and archwire deformation in traditional sliding mechanics.

**Additional file 2**: Video for virtual teeth movement and archwire deformation in miniscrew sliding mechanics.

## Data Availability

All data generated or analysed during this study are included in this published article and its supplementary information files.

## Abbreviations

FA: Facial axis
FEA: Finite element analysis
CBCT: Cone-beam computed tomography
PDL: Periodontal ligament
LAP: Low archwire plane
MAP: Middle archwire plane
HAP: High archwire plane
CR: Center of Resistance
